# Biphasic NMR of Hyperpolarized Suspensions—Real-Time Monitoring of Solute-to-Solid Conversion to Watch Materials Grow

**DOI:** 10.1021/acs.jpcc.3c04198

**Published:** 2023-09-21

**Authors:** Ertan Turhan, Christopher Pötzl, Waldemar Keil, Mattia Negroni, Karel Kouřil, Benno Meier, Javier Agustin Romero, Krzysztof Kazimierczuk, Ieva Goldberga, Thierry Azaïs, Dennis Kurzbach

**Affiliations:** † Institute of Biological Chemistry, Faculty of Chemistry, University of Vienna, Währinger Str. 38, Vienna 1090, Austria; ‡ University of Vienna, Vienna Doctoral School in Chemistry (DoSChem), Währinger Str. 42, Vienna 1090, Austria; § Institute of Biological Interfaces 4, Karlsruhe Institute of Technology, Egenstein-Leopoldshafen 76344, Germany; ∥ Institute of Physical Chemistry, Karlsruhe Institute of Technology, Karlsruhe 76131, Germany; ⊥ Centre of New Technologies, University of Warsaw, ul. Banacha 2c, Warsaw 02-097, Poland; # Sorbonne Université, CNRS, Laboratoire de Chimie de la Matière Condensée de Paris (LCMCP), 4, place Jussieu, Paris F-75005, France

## Abstract

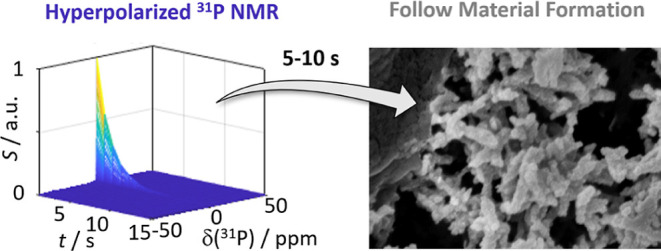

Nuclear magnetic resonance (NMR) spectroscopy is a key method for the determination of molecular structures. Due to its intrinsically high (*i.e.*, atomistic) resolution and versatility, it has found numerous applications for investigating gases, liquids, and solids. However, liquid-state NMR has found little application for suspensions of solid particles as the resonances of such systems are excessively broadened, typically beyond the detection threshold. Herein, we propose a route to overcoming this critical limitation by enhancing the signals of particle suspensions by >3.000-fold using dissolution dynamic nuclear polarization (*d*-DNP) coupled with rapid solid precipitation. For the proof-of-concept series of experiments, we employed calcium phosphate (CaP) as a model system. By *d*-DNP, we boosted the signals of phosphate ^31^P spins before rapid CaP precipitation inside the NMR spectrometer, leading to the inclusion of the hyperpolarized phosphate into CaP-nucleated solid particles within milliseconds. With our approach, within only 1 s of acquisition time, we obtained spectra of biphasic systems, *i.e.*, micrometer-sized dilute solid CaP particles coexisting with their solution-state precursors. Thus, this work is a step toward real-time characterization of the solid–solution equilibrium. Finally, integrating the hyperpolarized data with molecular dynamics simulations and electron microscopy enabled us to shed light on the CaP formation mechanism in atomistic detail.

## Introduction

The formation of solid calcium phosphate (CaP) is a highly important process in a plethora of timely industrial and academic applications, from bone replacements^1–4^ to heterogeneous catalysis^5^ to biomineralization.^6–8^ However, the nucleation and formation mechanisms typically remain out of the reach of existing characterization methods, impeding the derivation of rational design principles, albeit a timely need for functional materials with tailored properties.^9–11^ This critical shortcoming is due to the fact that the key player in high-resolution structure determination, nuclear magnetic resonance (NMR) spectroscopy, cannot access these processes, mainly due to two reasons: (i) these events proceed too fast (often within only a few seconds), compared to the typically long NMR acquisition times.^12^ (ii) During the precipitation processes preceding the formation of solids, dilute suspended particles coexist with dissolved ionic precursors. Solution-state NMR cannot detect both phases simultaneously as the highly diluted solid particles yield broadened signals of extremely low intensity, far beyond the detection limit. Recently, different methods have been proposed to monitor crystallization processes in aqueous solutions (*i.e.*, biphasic systems) using magic angle spinning (MAS) NMR. For example, CLASSIC NMR exploits the simultaneous measurement of both liquid-state and solid-state NMR spectra as a function of time to obtain information on the time evolution of both the solid phase and the solute species.^13^ A more recent approach is based on the freezing and solid-state NMR analysis of reactive solutions at low temperatures to trap transient species. Interestingly, the latter approach can be combined with dynamic nuclear polarization (DNP) to enhance sensitivity.^14^ These NMR approaches enable the recording of NMR fingerprints of both solid and solute species; however, the very first stages of crystallization are hardly accessible due to the limitation in time resolution that is induced by the limitation in sensitivity for CLASSIC NMR and how fast the solution can be frozen for the MAS DNP approach (typically several minutes).

Many recent examples, *e.g.*, by Simpson and co-workers in the context of biological substrates^15^ or Leskes and co-workers in the context of lithium-ion batteries,^16^ further showcase the current interest in solid– or solution–liquid interfaces and NMR methods to access biphasic systems.

Herein, we demonstrate that NMR analysis of biphasic solution/solid systems is possible, even for dilute (tens of mM) suspensions, using dissolution dynamic nuclear polarization (*d*-DNP).^17–19^ With *d*-DNP, we could boost NMR signals of solid CaP particles by over 3 orders of magnitude. The signal enhancement enabled the real-time NMR monitoring of the emergence of the solid particles in parallel with the consumption of solvated precursors at a sampling rate of 1 s^–1^.^20–22^ Capitalizing on the high intrinsic sensitivity of NMR to the chemical environment of individual nuclei and integrating it with molecular dynamics (MD) simulations and electron microscopy (EM), we could furthermore propose the chemical structure of the precursors and particles and thus shed light on the solution-to-solid conversion mechanism. While our earlier work^12^ showed that *d*-DNP can be used to monitor the conversion of free ions into solution-state self-assemblies, this approach is extended herein to enable hyperpolarized NMR of biphasic (solid/liquid) systems.

## Methods

### Dissolution DNP

For DNP, all samples were prepared similarly: 0.5 M K_2_HPO_4_ was dissolved in a mixture of glycerol-*d*_8_ and H_2_O in a volumetric ratio of 15:85. 4-Hydroxy-2,2,6,6-tetramethylpiperidine-*N*-oxyl (TEMPOL) was dissolved in the resulting solution at a concentration of 0.015 M. DNP was performed using the system described in ref (23) at a temperature of 1.3 K and a magnetic field of 6.7 T for ca. 5 h on a 150 μL volume sample. The best buildup kinetics were observed using continuous microwave irradiation at 188.048 GHz. Dissolution with 5 mL pressurized D_2_O, transfer, injection, and data acquisition were fully automated by a home-built prototype similar to the one described in ref (24), leading to injections of 300 μL of hyperpolarized sample in <2 s into the Shigemi NMR tube (without piston) waiting in the NMR spectrometer. To maintain the hyperpolarization during sample transfer, all fluid passages were sheltered with either Halbach magnets or pulsed solenoids. ^31^P signals were detected simultaneously using θ = 8° flip angles (corresponding to a 1 μs pulse at 42.7 W) for excitation with a repetition rate of 1 s^–1^ on a Bruker NEO 500 MHz spectrometer equipped with a BBFO Prodigy cryogenic probe. The acquisition time was 0.2 s, corresponding to 8192 data points. The pulse sequence was home-written and is available in the deposited data set (see Data Availability). All data were baseline-corrected and apodized by using an exponential window function after FT. All further information can be obtained from the deposited data sets.

For the mixing experiments, Shigemi NMR tubes (Shigemi Co., Ltd.) were prefilled with 150 μL of CaCl_2_ solutions (200 or 20 mM) buffered with 0.1 M 4-(2-hydroxyethyl)-1-piperazineethanesulfonic acid (HEPES), pH 8 in D_2_O. Upon injection of 300 μL, the resulting sample volume was 450 μL. This procedure resulted in a final phosphate concentration of 12.8 mM and a final CaCl_2_ concentration of either 67 or 6.7 mM.

Note that earlier studies showed an impact of the concentration of Ca^2+^ ions on the ^31^P resonance frequencies of the observed phosphate species. These studies were conducted with minimal buffer concentration (10 mM Tris buffer),^12^ such that the ion concentration exceeded the buffer capacity. In the present study, the buffer strength was thus chosen to be much higher to fully compensate the impact of Ca^2+^ and P_i_ ions, as both can act as effective acids/bases.

D_2_O of the HEPES buffer was used as a lock solvent. The temperature variations upon mixing were below 1 °C (see ref (25) for details on the used injection and mixing system.)

Repetition experiments yielded similar spectra to demonstrate the reproducibility of the results. All data can be downloaded from the data deposit specified in the Data Availability section.

The effective relaxation rates were determined by fitting the time-dependent line integral (determined using the “fitnlorentzian.m” function for the MATLAB program package) to monoexponential decay functions. The resulting errors are reported in Table 1. Note that the effective relaxation rate contained a contribution from signal loss due to continuous detection by a factor of sin^*n*^ (θ) (*n* being the number of detections), amounting to ca. 14% of the magnetization per detection. This effect is similar for all probed resonances. Taking into account that the sedimentation process of the solid particles was too slow to impact *R*_1,eff_, the rates reported in Table 1 can therefore be directly compared.

**Table 1:** Effective Relaxation Rates *R*_1,eff_ and Chemical Shifts δ(^31^P) of the Observed Phosphate Species

[Ca^2+^]/mM	species	*R*_1,eff_/s^–1^	δ(^31^P)/ppm
67	particle	0.56 ± 0.03	4.5
	PNS	1.34 ± 0.5	2.35
6.7	particle	0.51 ± 0.1	4.5
	PNS	0.65 ± 0.02	2.35
0	P_i_	0.25 ± 0.01	2.59

### Scanning Electron Microscopy

A similar procedure as outlined in ref (11) was performed. After the *d*-DNP experiments, the samples were removed from the spectrometer. The supernatant was removed from the suspension by centrifugation for 2 min at 14,000 rpm. The suspension was dissolved in 500 μL of H_2_O and again centrifuged at 14,000 rpm. Then, the pellet was carefully separated from the supernatant. This washing procedure was repeated three times. The final pellet was dissolved in 1 mL of H_2_O and diluted 1:10 to obtain a good dilution for the scanning electron microscopy (SEM) experiments (Zeiss Supra 55 VP).

Note that the final TEMPOL concentration after completion of the mixing process was 4.3 mM, which is on the order of Ca^2+^ and P_i_ concentrations. Hence, the influence of TEMPOL on the material formation cannot be excluded. Other radical concentrations tested (2.9 mM), yet, led to the same result.

### Molecular Dynamics Simulations

Calcium phosphate simulations were performed on a Workstation PC equipped with an AMD Threadripper PRO 5995WX processor (64*c*/128t) or an INTEL Core i9 12900 KS (16c/24T), 256GB DDR4-3200 MHz RAM, and an NVIDIA RTX 3090Ti GPU (driver version: 515.65.01/CUDA version: 11.7). The operating system was Rocky Linux 9 (Kernel version: 5.14) with the GCC/G++ compiler version of 11.2.

The GROMACS 2022.2 software package was used to set up and run the calcium phosphate simulation. The used force field was the all-atom additive CHARMM36 (July 2021 update) and was obtained from the MacKerell Web site.^26–30^ The TOPPAR files of HPO_4_^2–^ and H_2_PO_4_^–^ were obtained and converted to GROMACS file formats on the CHARMM-GUI Web site.^31–34^

The ions were placed in a cubic box with an edge length of 10 nm and solvated in water by using the SPC/E water model.

The simulation box contained 30 Ca^2+^ ions, 11 HPO_4_^2–^ ions, 7 H_2_PO_4_^–^ ions, 60 Cl^–^ ions, 29 Na^+^ ions, and 32,585 H_2_O molecules. Energy minimization was performed with the steepest descent algorithm. The V-rescale thermostat was used for NVT equilibration for a duration of 200 ps. After NVT equilibration, the NPT equilibration was performed with a Parrinello–Rahman barostat for 200 ps. The duration of the production run under the NPT conditions was 1000 ns.

### Solid-State NMR

A 200 mM CaCl_2_ solution was prepared in 0.1 M HEPES buffer (pH 8) with a total volume of 300 mL. A 500 mM P_i_ solution was prepared in a glycerol/H_2_O mixture in a volumetric ratio of 15:85, with a total volume of 24 mL, which was further diluted to obtain 20 mM P_i_ solution with the total volume of 600 mL.

The sample was prepared as follows: to a 200 mM CaCl_2_ solution (150 mL), 20 mM P_i_ solution (300 mL) was added fast and left under stirring for 10 min. Upon mixing both solutions, the formation of a white precipitate was observed. The white solid was collected by centrifugation for 4 min at 6000 rpm and then washed with 40 mL of water (three times by centrifugation using the same conditions).

The samples were packed inside an insert that fits a 4 mm rotor. ^31^P and ^1^H solid-state NMR experiments were performed in a 300 MHz field with an MAS frequency of 8 kHz.

Multivariate curve resolution (MCR): it should be noted that the signal-to-noise (SNR) ratio of the solid signal can be substantially improved using MCR. It is a chemometric method developed to recover information on pure components from data of complex mixtures.^35^ MCR can be applied to any kind of signal showing additive linear responses, which include all spectroscopic measurements. This procedure is particularly effective when the SNR approaches unity toward the end of the acquired times series (see Supporting Information Figures S11 and S12). To avoid the introduction of artifacts, the kinetic traces in Figure 2 have not yet been determined without MCR.

**Figure 1:**
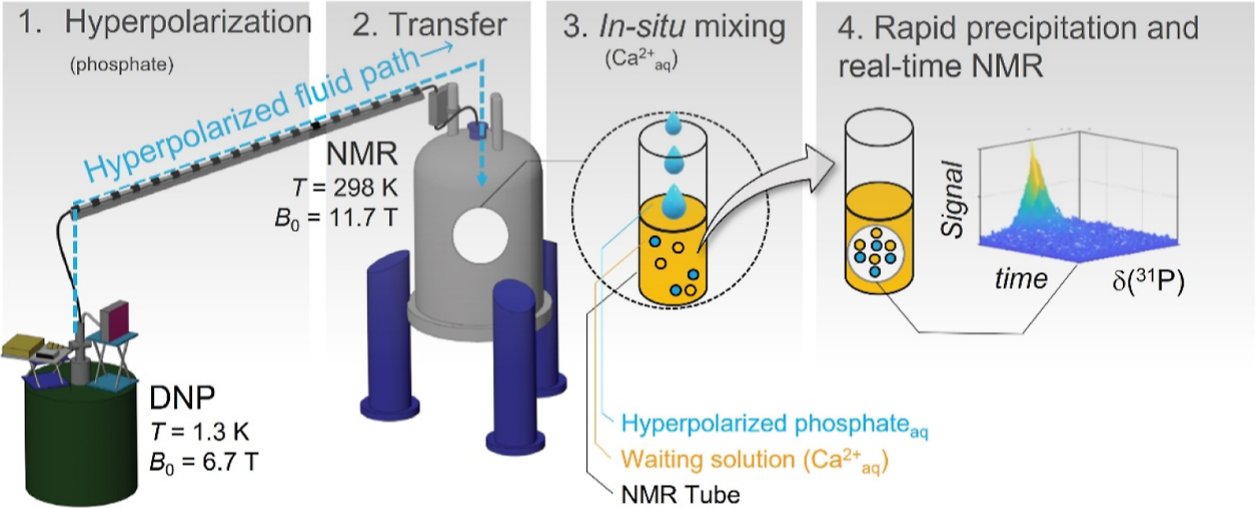
*d*-DNP approach to suspension NMR. 1. A frozen phosphate solution is hyperpolarized by DNP at *T*_DNP_ = 1.3 K and *B*_0_ = 6.7 T using microwave irradiation at 188.048 GHz. 2. The solution is dissolved and transferred to an NMR spectrometer. 3. It is mixed with a CaCl_2_ solution within the NMR tube. 4. Signal-enhanced real-time monitoring was achieved by recording a series of ^31^P NMR spectra using 8° flip angles. The inset shows an example of the obtained real-time data of a solid–liquid biphasic system.

**Figure 2:**
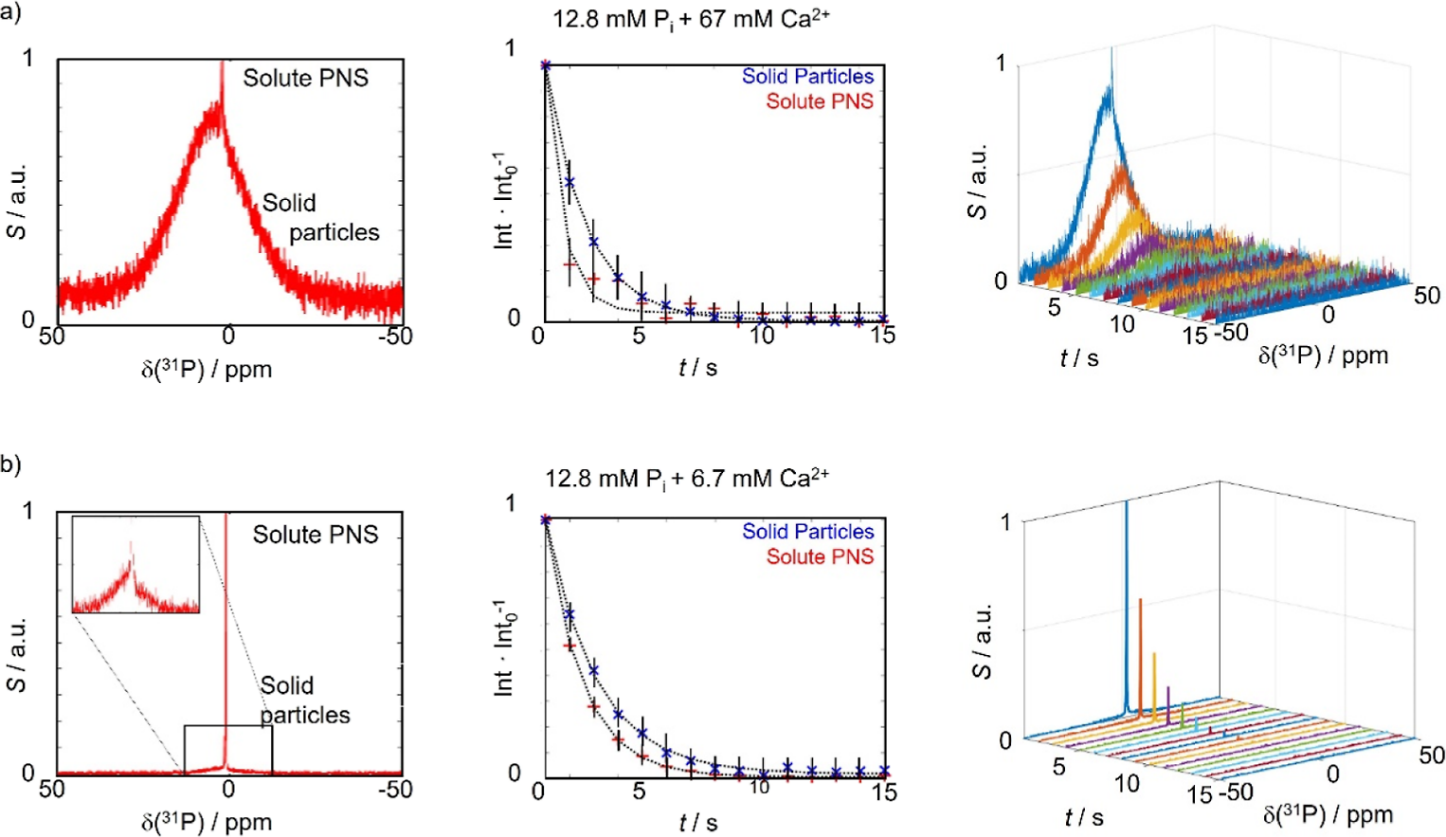
(a) ^31^P NMR spectrum obtained by *d*-DNP at high Ca^2+^ concentrations. The broad resonance of the solid particles (labeled solid) can be discerned from the narrow resonance of several solute transient species in fast chemical exchange (labeled PNS). The central panel shows the decay of the respective signal intensities (red and blue crosses) superposed with exponential fits (black dotted lines). The right panel shows the raw experimental data. Errors are indicated as black solid lines. (b) Same as in (a), but at a lower Ca^2+^ concentration. Fewer solid particles are produced, yet the line shape remains unchanged. Furthermore, the inset in the ^31^P spectrum highlights the solid-state contributions. If needed, the corresponding signal can be improved further by denoising procedures (Supporting Information).

## Results and Discussion

The presented methodology uses CaP as the main model system due to its widespread use and interest.^1–4,36,37^ The general concept of our approach is to spin-hyperpolarize, *i.e.*, signal-enhance an NMR-active component of the final solid to be studied *ex situ* in a dedicated DNP apparatus (the used system is described in detail in ref (23)). Herein, we aim to employ inorganic phosphate (P_i_) as a reporter nucleus. To this end, pretreated, *i.e.*, hyperpolarized P_i_ is rapidly dissolved after DNP at 1.3 K for >1 h, transferred to an NMR spectrometer, and mixed *in situ* with a CaCl_2_ solution waiting in the NMR tube. Using a prototype for rapid sample injection as described in ref (24) the mixing process was completed within 1.5 s. Subsequently, signal acquisition by a series of small flip-angle pulses was initiated. At 298 K, pH 8, and Ca^2+^ and P_i_ concentrations of>5 and12.8 mM, respectively (as used herein), the precipitation of solid calcium phosphate particles from aqueous buffers starts instantaneously and, thus, proceeds during the acquisition period.

Due to the *d*-DNP signal boost, the ^31^P solid-state resonances could be recorded within a single scan during CaP formation, enabling real-time process monitoring. The ″*in situ* precipitation” DNP approach^12^ is sketched in Figure 1.

The resulting spectra (recorded immediately after completion of the mixing step) at varying Ca^2+^ concentrations (67 and 6.7 mM, respectively, at a fixed P_i_ concentration of 12.8 mM) are shown in Figure 2a,b (left panels). For both cases, the heavily broadened resonance of the solid particles concomitant with their nucleation can be identified and superposed with the sharp signal of the solute precursors (labeled solid particles and solute PNS, prenucleation species; *vide infra*). The signal enhancements were determined to be ε ≈ 3000 after precipitation (4000 in the absence of CaCl_2_; reference data for *d*-DNP of P_i_ in the absence of any Ca^2+^ ions can be found in Supporting Information Figure S1). It should be noted that acquiring the reference signal by conventional NMR in thermal equilibrium to calculate the enhancement factor is complicated because of the long acquisition times during which the solid particles sedimented and displayed prohibitively weak signal strengths. Furthermore, the effects of size distribution on the reference signal intensities could bias the determined enhancements.

Figure 2 further shows that the signal intensity ratio between solid and solute phosphate species depended on the availability of Ca^2+^ ions. If Ca^2+^ is in large excess (67 mM) relative to P_i_, an intense solid line shape is observed where ca. 99% of the PNS were converted into the solid, as derived from the ratio of the respective line integrals in the first detection. When Ca^2+^ is the limiting reactive (6.7 mM), a slower and/or incomplete conversion into solid CaP is observed, resulting in a less intense solid-state resonance relative to the liquid-state signal, where only 41% of the initial PNS were converted.

**Figure 3:**
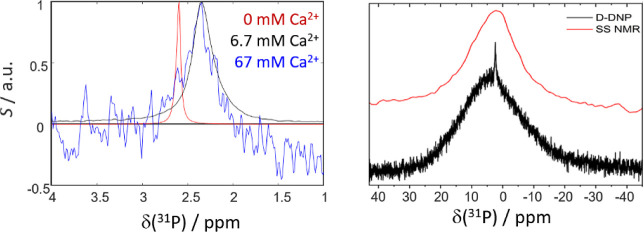
(a) Zoom onto the sharp resonance of ^31^P NMR spectra obtained by *d*-DNP at different Ca^2+^ concentrations. The resonance in the presence of CaCl_2_ (black, blue) is Ca^2+^ concentration-independent, broader than that of free phosphate (red), and shifted upfield by ca. 0.26 ppm. (b) Static solid-state NMR spectrum (red) compared to the spectrum obtained by *d*-DNP (black). The solid-state spectrum was recorded after centrifugation and concentration of the particle suspension resulting from the *d*-DNP experiment and removal of the supernatant.

In contrast, the line shape of both solute and solid species remained constant, independent of the Ca^2+^ concentration (see Figures 3a and S2).

Due to the high signal enhancements and the resulting short acquisition times, the suspensions could be monitored with a sampling rate of 1 s^–1^. However, as spin-hyperpolarization is a non-equilibrium state, the signal enhancements decay with an effective longitudinal relaxation rate *R*_1,eff_Figure 2a,b (central panels) shows the time traces of the resonance integrals for both probed Ca^2+^ concentrations (sharp and broad components labeled solute PNS and solid particles). The respective time series of spectra are shown in the right panels of Figure 2a,b.

The signal of the solvated component decayed faster than that of the precipitate for both probed Ca^2+^ concentrations (the different *R*_1,eff_ rates are listed in Table 1, and for error discussion, see the Methods section). In contrast, solvated P_i_ in the absence of any Ca^2+^ relaxes 3- to 4-fold slower (Figure S1). To interpret this observation, we must consider that we monitored the CaP system during solidification. Hence, solvated precursor species are consumed and converted to solid CaP throughout the detection period. Thus, the differential signal decay rates can be traced back to a rapid conversion of solvated phosphate species into solid particles, leading to an apparent faster *R*_1,eff_ of the latter compared to pure solute Pi. Reducing the CaCl_2_ concentration from 67 to 6.7 mM, this trend is still observable, yet the PNS consumption is slowed down as fewer Ca^2+^ ions ([Ca^2+^] < [P_i_] in this case) are available to drive the solidification process.

Interestingly, the line width of the observed solute species is ca. three times larger than that of free phosphate in solution. The chemical shift of the solute also deviates from that of free P_i_ (Figure 3 and Table 1). Hence, we expect that in the presence of Ca^2+^, the solute phosphate species are not mere free P_i_ but more likely free P_i_ in exchange with other (potentially many) Ca^2+^-interacting species. This observation aligns well with the description of nonclassical nucleation pathways for CaP phases^8,12,37–39^ where nanometric solvated transient calcium phosphate species precede the formation of solid CaP. These species were denoted as “prenucleation species” (PNS).^12,37,40,41^ These species feature broadened ^31^P resonances, much like the signals in Figure 3a,^12^ due to their high molecular weights and consequently slow tumbling. Note that the conditions chosen for the herein showcased proof-of-concept of biphasic hyperpolarized NMR are much different (buffer composition, pH, and ionic strength) from those reported earlier,^12^ which led to the observation of free P_i_ and PNS-bound P_i_ in slow exchange in solution. Under the present conditions, we observe only one solute resonance, which likely results from an exchange of averages between several PNS species and free P_i_. However, as the observed PNS signal is concentration-independent, the contribution of free P_i_ cannot be significant.

To further investigate the molecular structure of the solute species, we performed additional computational analyses. The combination of *d*-DNP with molecular dynamics (MD) simulations can help in obtaining structural information and complement the hyperpolarized spectral fingerprints. In particular, when the ^31^P resolution obtained with *d*-DNP does not allow for a detailed structural analysis of the species formed during CaP transformation, the combination with MD simulations is advantageous, as these can provide insights into these species' structures.

**Figure 4:**
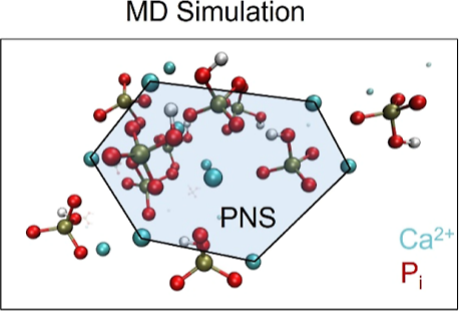
Snapshot of an MD simulation at high Ca^2+^ concentrations after 1000 ns. At the high concentration used, PNS forms already within this time. The PNS adopts a loose hexagonal arrangement. Detailed analyses of the MD data can be found in the Supporting Information.

An MD simulation of a solution of Ca^2+^ and P_i_ at the concentrations present immediately after mixing in the *d*-DNP experiments (*T* = 25 °C, pH = 8, constant ionic strength, for the experiments and simulations) confirmed the preferential formation of stable Ca^2+^ and phosphate aggregates. Figure 4 shows that the simulated ionic self-assembly, *i.e.*, the PNS, started forming in explicit water after ca. 500 ns and completed within ca. 700 ns (Supporting Information Figures S7 and S8). Interestingly, these self-assemblies loosely adopt a hexagonal structure (Figure 4). After formation, they remained stable throughout the entire MD run. It was recently proposed^10^ that the precursors formed in solution likely predetermine the molecular structure of the resulting solid upon aggregation. In this context, this stable hexagonal arrangement found in solution could be a remnant structural motif in the solid ionic arrangement. Notably, such PNS have recently gained attention due to their potential use as bioinorganic qubits.^42,43^

Further note that the MD simulations only correspond to the first moments, *i.e.*, 1 μs after mixing the Ca^2+^ and P_i_ solutions. Hence, they report on only the very early stages of CaP formation, *i.e.*, the initial clustering. The first spectrum was recorded 1 s after mixing in the DNP experiments. Hence, the immediate presence of a signal deviating from that of free P_i_ in solution is consistent with the rapid clustering in the MD simulations.

**Figure 5:**
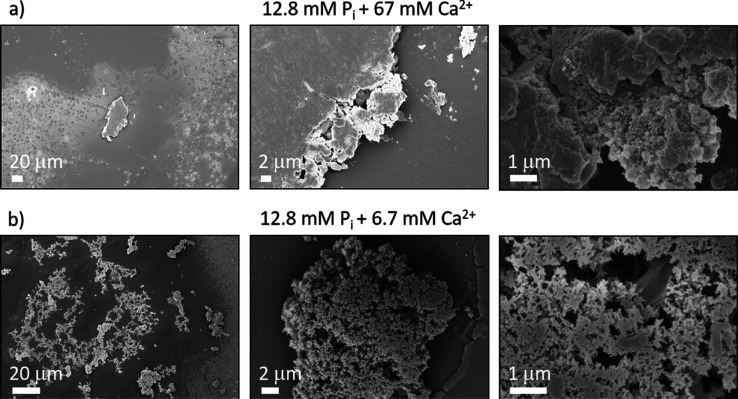
SEM images of the CaP particles formed in the *d*-DNP experiments. In both cases, the final particle size exceeds 10 μm, explaining the detection of the solid-state type resonances in the particle suspensions. Further SEM images can be found in the Supporting Information Figures S9 and S10.

Finally, we investigated solid particles by SEM. The electron micrographs are shown in Figures 5, S9, and S10. We observed solid particles with sizes >10 μm. At the reported magnification, the EM images provide information about the morphology of aggregates and not about the molecular structure of the crystallites (*i.e.*, the crystalline phase). The solid-state morphologies resulting from the two probed concentrations differed. While lower Ca^2+^ concentrations led to the observation of porous dendrite-like particles, higher Ca^2+^ concentrations led to nonporous platelets. As all involved PNS showed similar NMR signatures over the entire acquisition time, we conclude that the association and dehydration kinetics, not only the structures of preformed PNS, primarily orchestrate this difference in solid-state density.

Additional solid-state MAS NMR experiments showed that hydroxyapatite (HA) is the final CaP phase that forms under both experimental conditions. However, we also show that HA is formed from a transient phase, namely octacalcium phosphate (OCP), as seen after 10 min of reaction (Supporting Information Figures S3–S6). Hence, we infer that the real-time *d*-DNP experiments accessed the dynamic equilibrium between solvated PNS and the nucleated solid, *i.e.*, the solid transient phase (most likely the OCP), which later transformed into HA.

## Conclusions

In conclusion, we present a methodology to characterize dilute suspensions of solid particles in equilibrium with solution-state precursor assemblies by hyperpolarized NMR spectroscopy in real time. Integration with MD simulations and EM enabled insights into the material formation processes, showcased herein with the example of the widely used CaP. We found that under our conditions the formation of HA is initiated by PNS formation, followed immediately by the nucleation of the solid precursor. Solvated PNS adopt a stable atomistic structure over time that is of ca. 2 nm in size and aggregate in a secondary event to produce bigger nucleated particles that later on transform into HA.

The constant chemical shift of the solvated species suggests that the structure of the Ca^2+^ coordination sphere around the phosphate ions does not change with the Ca^2+^ ion concentration. However, it cannot be excluded that several PNS of different sizes (but potentially locally similar structures) coexist in different amounts that exchange with free P_i_. Furthermore, the simulated precursors displayed features that could be a remnant structural motif in the solid ionic arrangement.

Interestingly, the kinetics of PNS aggregation resulted in the formation of solid CaP with different densities and solid-state morphologies—an observation made possible with the presented methodology and pointing toward the possibility of rationally controlling solid-state morphologies *via* their solution-state precursors.

As our approach to material formation monitoring is based on time-resolved NMR acquisition, applications to other systems housing NMR-active nuclei should be readily conceivable. This might be particularly useful, *e.g.*, in applications that aim to tailor material surface properties, such as the design of heterogeneous catalysts, qubits, or greenhouse gas scavengers.

## Data Availability

All raw NMR data can be found under DOI 10.5281/zenodo.8325083. The MD trajectories can be downloaded from https://phaidra.univie.ac.at/o:1799745, https://phaidra.univie.ac.at/o:1800178, https://phaidra.univie.ac.at/o:1800256.

## Supporting Information Available

Additional *d*-DNP, solid-state NMR, and EM data; MD distance and RMSD trajectories; and details and results of MCR (PDF)
