# Supplementary material for: Biphasic NMR of Hyperpolarized Suspensions—Real-Time Monitoring of Solute-to-Solid Conversion to Watch Materials Grow
Source: J Phys Chem C Nanomater Interfaces. 2023 Sep 21;127(39):19591–8. doi: 10.1021/acs.jpcc.3c04198 (PMC10561236; doi:10.1021/acs.jpcc.3c04198)
Supplement: Supplementary file 1 — jp3c04198_si_001.pdf [file jp3c04198_si_001.pdf]

Biphasic NMR of Hyperpolarized Suspensions – Real-Time Monitoring of Solute-to-Solid Conversion to Watch Materials Grow.

*Ertan Turhan[1,2], Christopher Pötzl[1,2], Waldemar Keil[1], Mattia Negroni[1], Karel Kouřil[3], Benno Meier[3,4], Javier Agustin Romero[5], Krzysztof Kazimierczuk[5], Ieva Goldberga[6], Thierry Azaïs[6], Dennis Kurzbach[1,2]**

[1]Institute of Biological Chemistry, Faculty of Chemistry, University of Vienna, Währinger Str. 38, 1090 Vienna, Austria
[2]University of Vienna, Vienna Doctoral School in Chemistry (DoSChem), Währinger Str. 42, 1090 Vienna, Austria
[3]Institute of Biological Interfaces 4, Karlsruhe Institute of Technology, 76344 Egenstein-Leopoldshafen, Germany
[4]Institute of Physical Chemistry, Karlsruhe Institute of Technology, 76131 Karlsruhe, Germany
[5]Centre of New Technologies, University of Warsaw, ul. Banacha 2c, 02-097 Warsaw, Poland
[6]Sorbonne Université, CNRS, Laboratoire de Chimie de la Matière Condensée de Paris (LCMCP), 4, place Jussieu, F-75005 Paris, France
**corresponding author: dennis.kurzbach@univie.ac.at*

-Supporting information-

Liquid state measurements

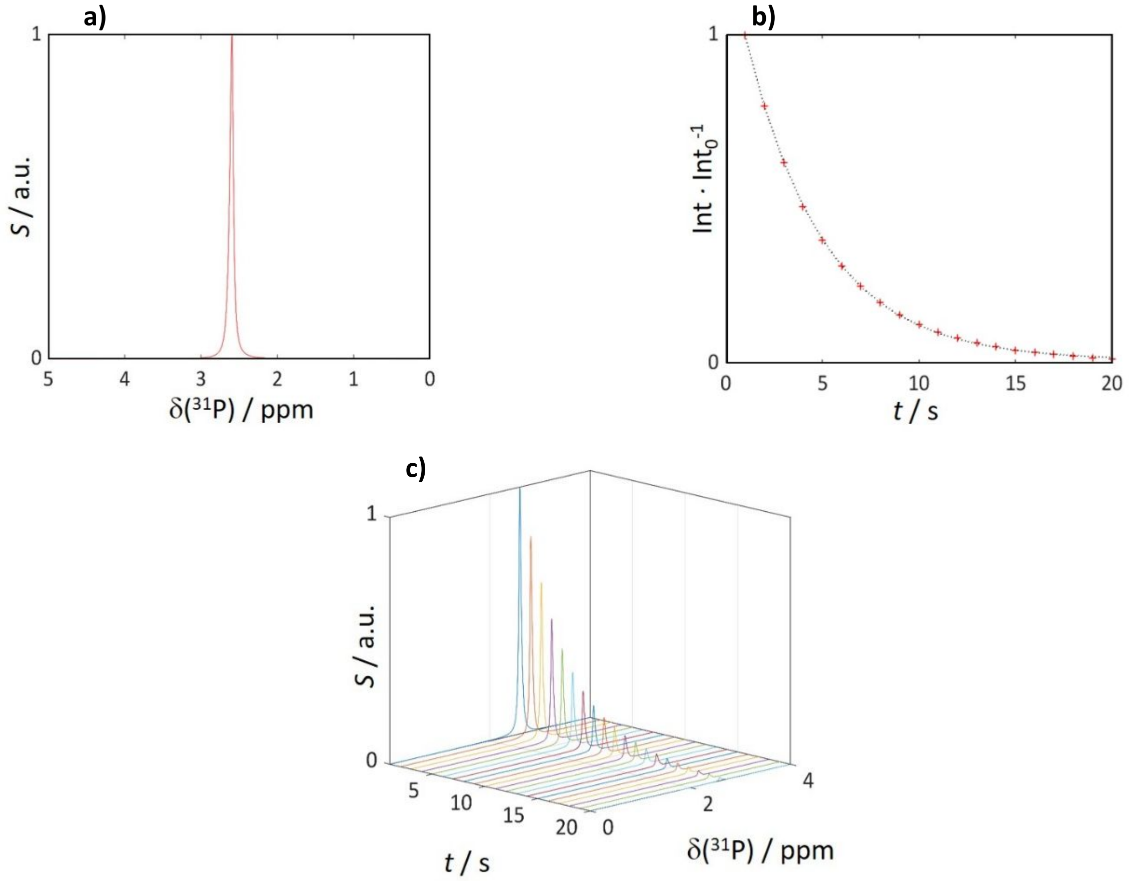

Figure S1. a) ^{31}P NMR spectrum obtained by *d*-DNP of 0.5 M P_i in the absence of any $CaCl_2$. One single resonance around 2.59 ppm indicates the presence of neither solid particles nor solute PNS. b) Display of

the decay of the signal intensity (red) superposed with exponential fits (black dotted lines). c) Raw experimental data.

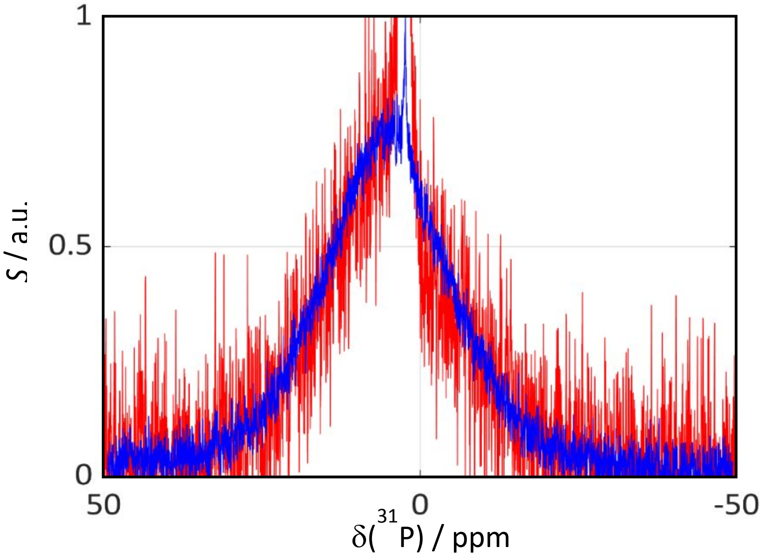

Figure S2. a) ^{31}P NMR spectrum obtained by *d*-DNP of both approaches (6.7 mM and 67 mM). The resonance of the solid particles at 4.5 ppm at 6.7 mM $CaCl_2$ (red) superimposed with the solid particles obtained at 67 mM $CaCl_2$ (blue).

Solid state measurements

The sample for the ssNMR analysis was prepared using the same concentrations as described in the experimental section. To a 200 mM [Ca] solution (150 mL), a 20 mM [Pi] solution (300 mL) was added fast and left under stirring for 10 mins. Upon mixing both solutions, the formation of a white precipitate was observed. The white solid was collected by centrifugation for 4 minutes at 6000 rpm, and then washed with 40 mL water (three times by centrifugation using the same conditions).

ssNMR was performed on the wet sample (after the last washing step) and an overnight freeze-dried solid sample. All experiments were performed as reported earlier.[1]

1. Weber, E. M. M.; Kress, T.; Abergel, D.; Sewsurn, S.; Azais, T.; Kurzbach, D., Assessing the Onset of Calcium Phosphate Nucleation by Hyperpolarized Real-Time NMR. *Anal Chem* **2020,** *92* (11), 7666-7673.

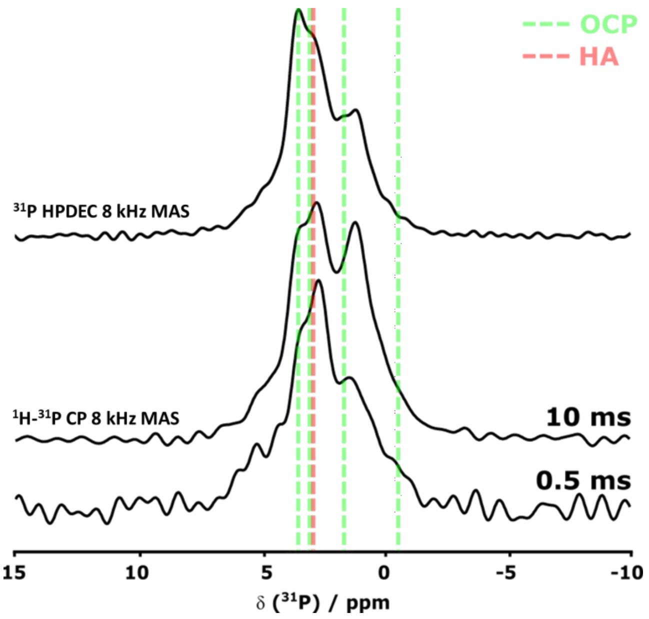

Figure S3. ^{31}P direct polarization (DP) and ^{1}H-^{31}P CP MAS ssNMR experiments on the fresh wet sample resulting solid showed multiple peaks with maxima at 3.5-3.6, 2.9 and 1.1 ppm. Experiments were recorded with recycle delay (RD) of 120 s with 96 number of scans (NS) for DP spectrum and RD = 3.5s, NS = 64 and contact time CT of 0.5 and 10 ms for CP MAS spectra.

^{31}P DP and CP MAS spectra display multiple resonances compatible with a mixture of HA and OCP phases. Red and green dashed lines indicate the expected positions of the ^{31}P resonances from HA and OCP (corresponding to P4/P1, P2 and P6/P3/P5 phosphorous sites from the OCP structure according to Davies *et al.* DOI: 10.1021/ja3017544).

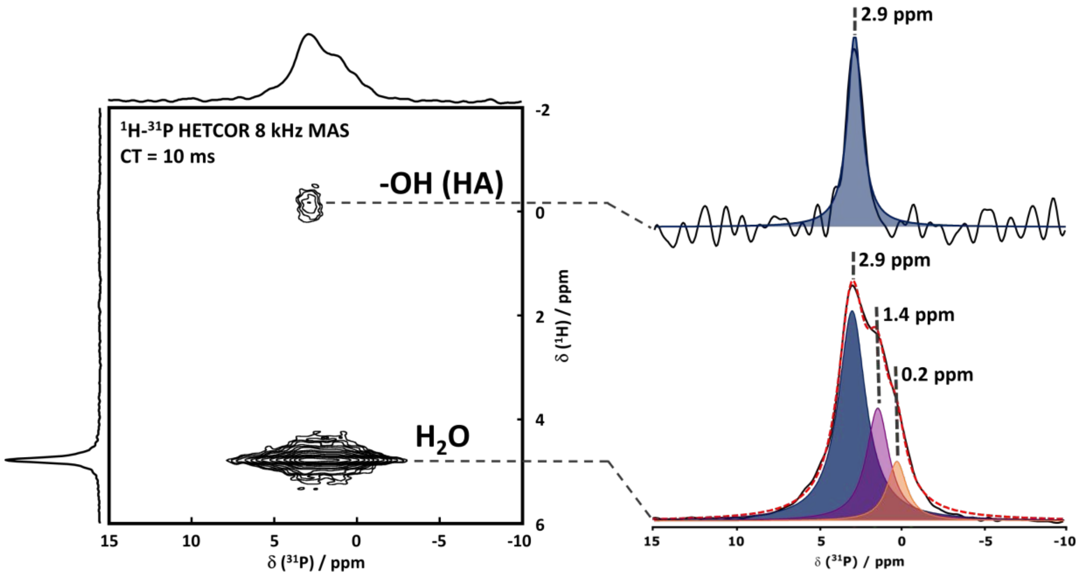

Figure S4. 2D ^{1}H-^{31}P HETCOR ssNMR experiment with ^{31}P spectra extracted from slices along 0 and 4.8 ppm in ^{1}H dimension. The resulting spectrum was recorded using a CT of 15 ms.

The 2D HETCOR spectrum further confirms the presence of two distinct phases in the sample: (i) HA displaying the characteristic OH^- contribution at $\delta(^{1}H)$ = 0 ppm and (ii) possibly immature and hydrated OCP at $\delta(^{1}H)$ = 4.8 ppm as the 2D spectrum is very similar to Fig. 5 in Robin et al. DOI : 10.1039/C9CE01972C, where the transformation of OCP into HA is monitored through *ex situ* ^{31}P ssNMR and *in situ* RAMAN. However, the contribution of brushite at $\delta(^{31}P)$ = 1.4 ppm cannot be totally excluded.

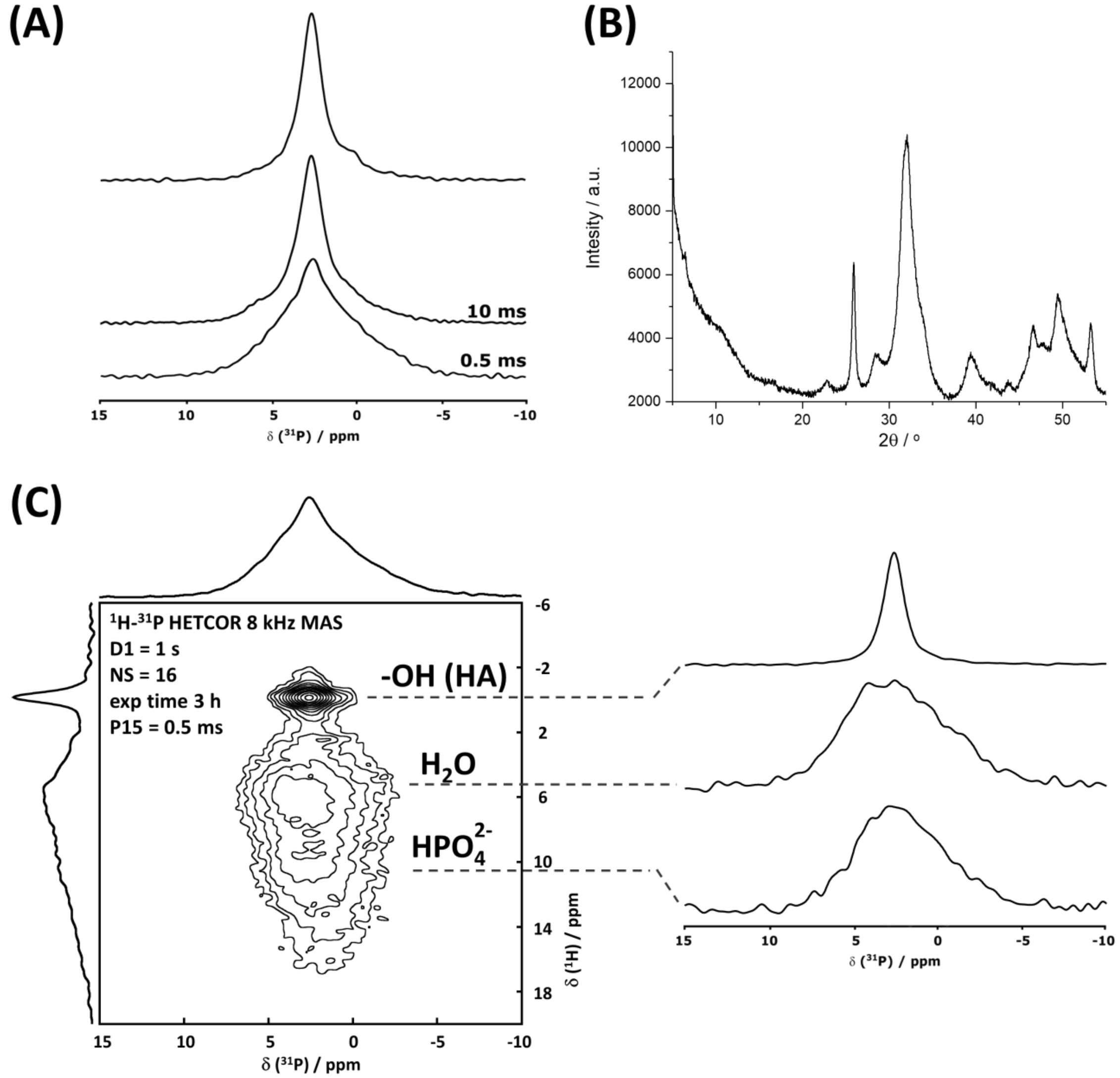

Figure S5. Solid-state NMR analysis performed on a freeze-dried sample. (A) ^{31}P DP and ^{1}H-^{31}P CP MAS experiments and (C) 2D ^{1}H-^{31}P HETCOR experiment with ^{31}P spectra extracted from slices along -OH (-0.1 ppm), H_2O (5.4 ppm) and HPO_4^{2-} (9.8 ppm) contribution in ^{1}H dimension. The resulting 1D spectrum was recorded with RD = 120 s with NS = 96 for the DP spectrum and RD = 1s, NS = 64 and contact time CT of 0.5 and 10 ms for the CP MAS spectra. 2D was recorded using a CT of 0.5 ms. (B) Powder XRD pattern of the same sample recorded in angular range (2Θ) of 5-55°. The given diffraction pattern corresponds to that of HA.

The ^{31}P NMR spectra show a peak centered at 2.6 ppm, corresponding to the HA phase as the main contribution suggesting the transformation of OCP transient phase into HA upon freeze drying. The 2D ^{1}H-^{31}P HETCOR reveals the presence of an additional broad contribution corresponding to phosphates close to water molecules and HPO_4^{2-} groups in agreement with the presence of an amorphous layer around HA particles.

The samples taken directly from the d-DNP and EM experiments further confirmed the predominant contribution of HA. A 1D ^{1}H-^{31}P CP experiment was performed with 2 and 10 ms contact times. Both show the same main peak between 2.8 and 2.9 ppm (Figure S7).

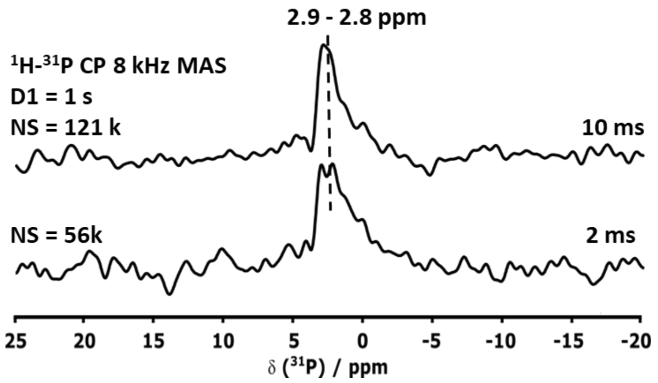

Figure S6. ^{1}H-^{31}P CP ssNMR experiments of the dried sample.

Molecular Dynamics Simulations

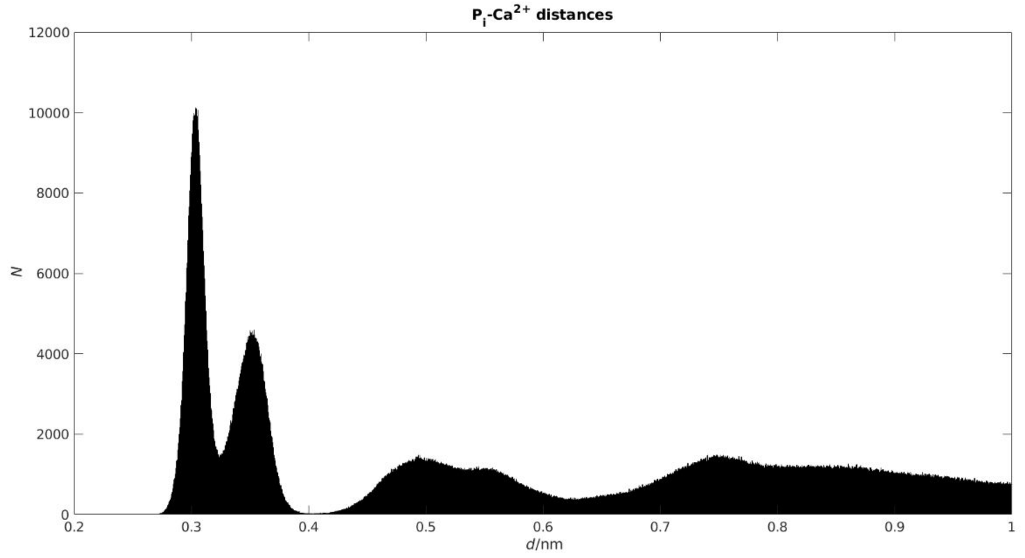

Figure S7: Histogram of the distribution of distances between all Ca^{2+} ions and phosphorus atoms over the simulation trajectory. The sharp distance distributions show that the PNS configuration is well-defined and preserved throughout the entire simulation run of 1 μs and that most P_i molecules embedded in PNS.

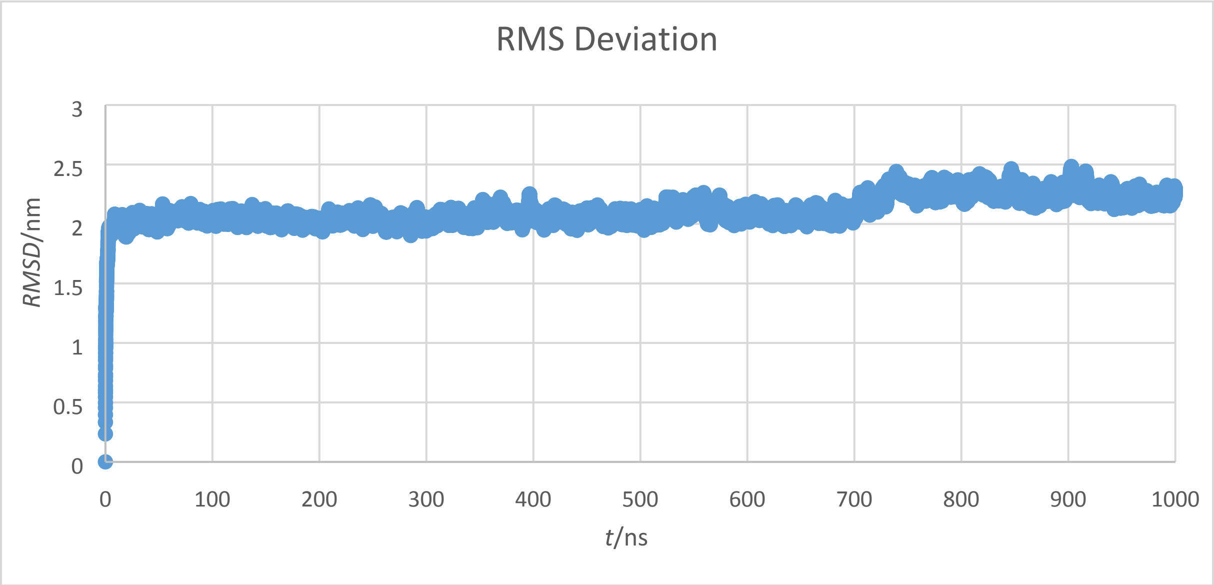

Figure S8: RMSD between all Ca^{2+} ions, HPO_4^{2-} ions and $H_2PO_4^-$ ions PNS form after 700 ns

Scanning electron microscopy measurements

10 µL of the obtained particle suspension (Experimental Section: Scanning Electron Microscopy) were applied onto ThermanoxTM coverslips and dried on air for two days. Subsequently, the coverslips were gold covered under high-vacuum (Bal-Tec SCD 005). Images were obtained with a Zeiss SEM supra 55 VP at 5 kV using the InLense and Everhart Thornley (SE2) detectors.

<u>67 mM $CaCl_2$ approach</u>

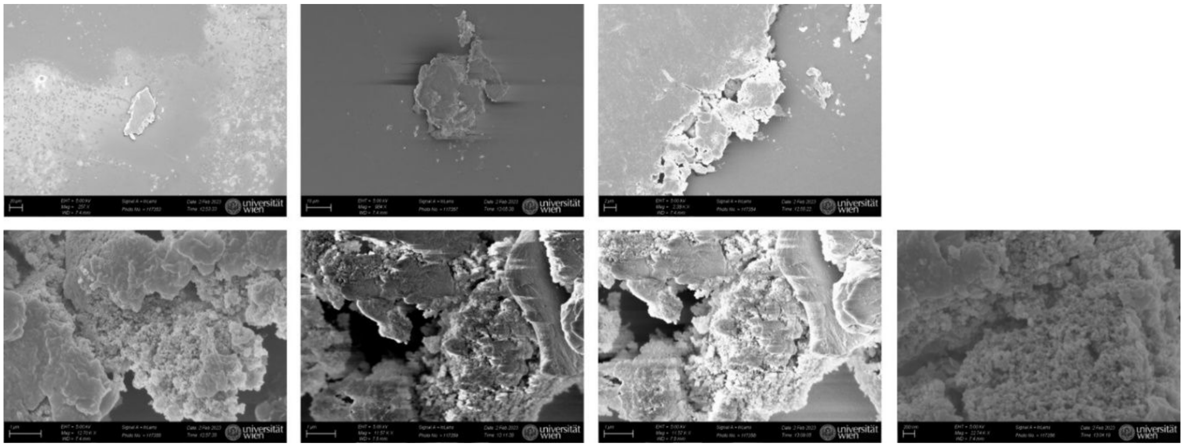

Figure S9. SEM images of the solid particles emerged during the experiments using the 67 mM $CaCl_2$ approach. These images are shown in increasing magnification from 20 µm to 200 nm.

<u>6.7 mM $CaCl_2$ approach</u>

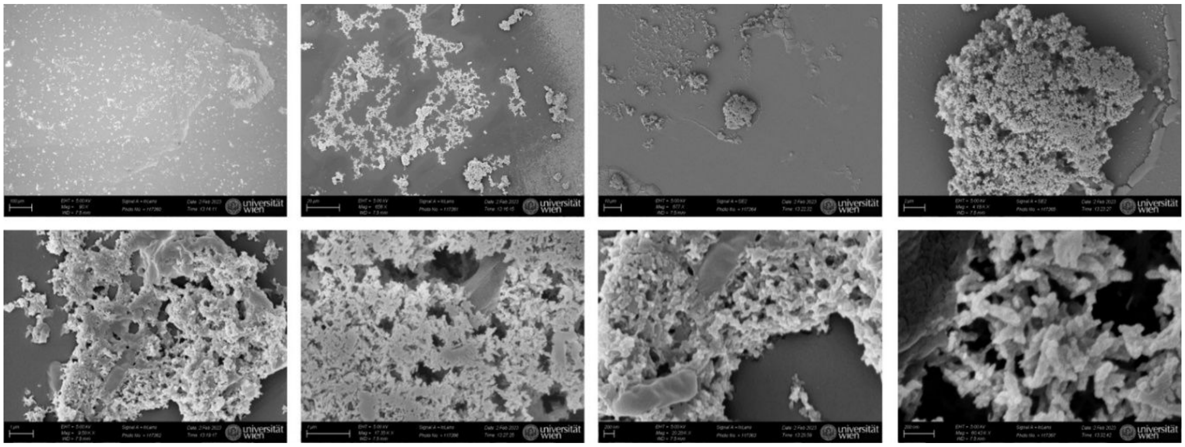

Figure S10. SEM images of the solid particles emerged during the experiments using the 6.7 mM $CaCl_2$ approach. These images are shown in increasing magnification from 100 µm to 200 nm.

MCR

The series of NMR signals were denoised using Multivariate Curve Resolution (MCR). MCR is a chemometric method developed to recover information of pure components from data of complex mixtures [1]. MCR can be applied to any kind of signals showing additive linear responses, which includes all spectroscopic measurements. Using the standard nomenclature found in the literature [2], MCR decomposes the data matrix D into a concentrations matrix C and a spectra matrix S, plus a residuals matrix E which should contain only noise:

$$D = CS^T + E$$

For the case of series of 1D NMR time domain signals, the matrix C contains the amplitude profiles of the NMR signals (i.e. time evolution of signals amplitudes), and the matrix S contains the time domain signals of the pure components. The factorization is obtained by means of the alternating least-squares approach. At the k-th iteration the values of C_k and S_k are computed as:

$$S_k^T = C_{k-1}^+ D$$

$$C_k = DS_k^+$$

$$E_k = D - C_k S_k^T$$

where "+" denotes the Moore-Penrose pseudoinverse. It is worth mentioning that the MCR method has been used once before in NMR for denoising 2D signals of solid-state measurements [2]. The common practice for obtaining the initial guess for the amplitude profiles (C_0) is to use the purest variable algorithm [3]. However, in our case, we decided to use the Singular Value Decomposition (SVD) for the estimation of C_0 at the cost of a negligible difference in the final results, but with a great simplification of the algorithm. The SVD decomposes D into

$$D = U\Sigma V^T$$

Then

$$C_0 = U\Sigma$$

The SVD is always the first step in the MCR method, since looking at the number of significant singular values of D is a good way of estimating the correct number of of pure components that should be retrieved

from the data. Although, the general practice is to apply the method repeatedly while varying the number of singular values used until the Lack-of-Fit drops below an acceptable value [4]. For denoising NMR signals, we used to the following criteria:

argmin{ norm[std(D-D') - std(spectral_noise)] }

where D' is the MCR reconstructed data matrix and 'spectral_noise' is the noise in the original spectra. Since our goal is to remove noise from the input matrix D, the standard deviation of the residual matrix E should resemble the standard deviation of the spectral noise. A simplified version of our Python code is available in the Supporting Information, but there are also other options available for Matlab [4]

[1] Tauler, Roma. "Multivariate curve resolution applied to second order data." *Chemometrics and intelligent laboratory systems* 30.1 (1995): 133-146
[2] Bruno, Francesco, et al. "Multivariate Curve Resolution for 2D solid-state NMR spectra." *Analytical chemistry* 92.6 (2020): 4451-4458
[3] Windig, Willem, and Jean Guilment. "Interactive self-modeling mixture analysis." *Analytical chemistry* 63.14 (1991): 1425-1432.
[4] Jaumot, Joaquim, et al. "A graphical user-friendly interface for MCR-ALS: a new tool for multivariate curve resolution in MATLAB." *Chemometrics and intelligent laboratory systems* 76.1 (2005): 101-110

We present here a simplified version of the code used for denoising. The input file for the code is the Bruker ser file. To use the code first process the series of NMR signals in TopSpin but without zero filling. The apply IFT in the Fourier dimension and execute the genser command. Save the new signal in a folder and give this folder path as input in the code. The genser command removes digital filter effect that is often present in NMR signals.

```
import numpy as np
from numpy.linalg import pinv
import matplotlib.pyplot as plt

N = 512     # Grid size F1
sz = 8192   # Grid size F2

file = open('path_to_ser_file', 'rb')
fid = np.fromfile(file, dtype=np.int32).reshape((N, 2*sz))
fidmo = fid[:, ::2] + 1j*fid[:, 1::2]
X = np.concatenate((np.real(fidmo), np.imag(fidmo)), axis=1)
u, s, vh = np.linalg.svd(X, full_matrices=False)

SVs = 4     # Number of Singular Values to truncate the SVD decomposition
est = np.matmul(u, np.diag(s))
C = est[:, :SVs]
max_iter = 20
threshold = 5
LOF = 100
iter = 0

while LOF > threshold and iter < max_iter:
    S = np.matmul(pinv(C, 0.1), X)
    C = np.matmul(X, pinv(S, 0.1))
```

```
    D = np.matmul(C, S)
    E = X - D
    LOFi = 100 * np.sqrt(np.sum(np.square(E)) / np.sum(np.square(X)))
    if LOFi < LOF:
        LOF = LOFi
        iter = 0
    iter = iter + 1
    print(iter, 'LOF =', LOF)

if iter == max_iter:
    print('STOP:', max_iter, 'iterations without improvement in Lack of Fit.')
else:
    print('STOP: Lack of Fit below', threshold, '% threshold.')

out = np.zeros(D.shape, dtype=np.int32)
out[:, ::2] = D[:, :sz]
out[:, 1::2] = D[:, sz:2*sz]
out = np.reshape(out, (2*sz*N, 1))

out_file = open('path_for_output_ser_file', 'wb')
out.tofile(out_file)
```

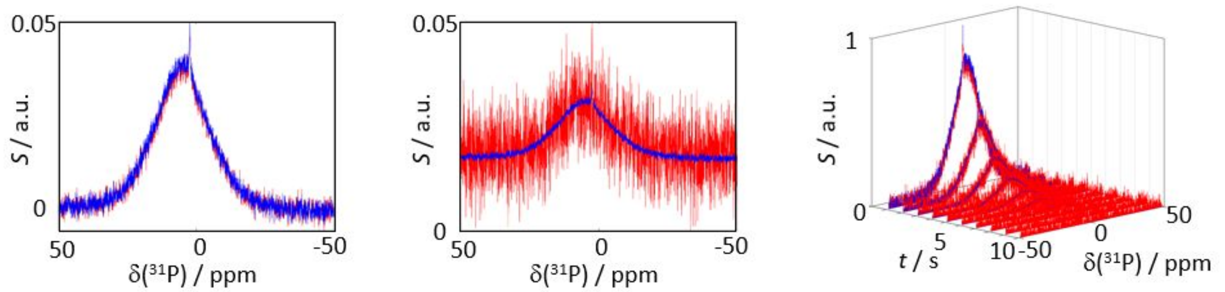

Figure S11. Original (red) and denoised by MCR (blue) spectra for the case of 67 mM Ca^{2+} ion concentration. Left: Frist spectrum of the time series. Center: 8th spectrum of the time series. Right: Full time series data.

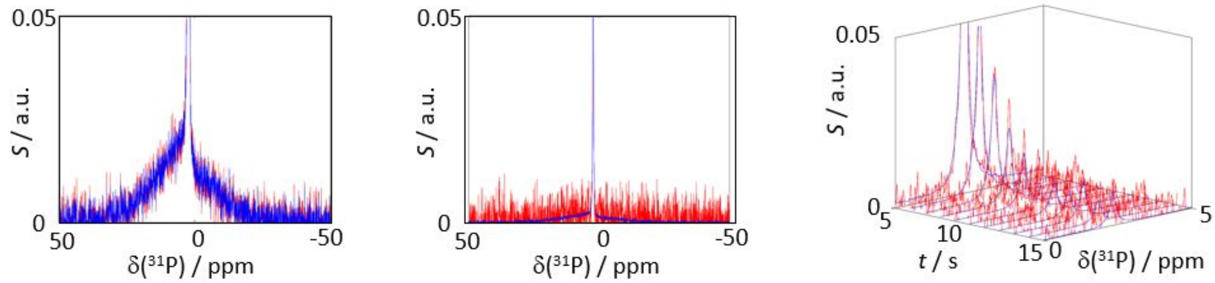

Figure S12. Original (red) and denoised by MCR (blue) spectra for the case of 6.7 mM Ca^{2+} ion concentration. Left: Frist spectrum of the time series. Center: 8th spectrum of the time series. Right: Full time series data.
